# Multi-omics integration of the vaginal microbiome and inflammatory proteome for non-invasive prediction of infertility: a machine learning approach

**DOI:** 10.3389/fcell.2026.1927235

**Published:** 2026-09-11

**Authors:** Yangkun Feng, Yu Zhang, Dandan Chen, Aifen Wang, Zhenxing Liu, Yun Zhang, Ruixi Yu

**Affiliations:** 1 Department of Reproductive Medicine Center, Wuxi Maternity and Child Health Care Hospital, Wuxi, China; 2 Department of Reproductive Medicine Center, The Affiliated Wuxi Maternity and Child Health Care Hospital of Jiangnan University, Jiangnan University, Wuxi, China; 3 Jiangnan University Wuxi School of Medicine, Wuxi, China; 4 Department of Gynecology, Wuxi No 2 People’s Hospital, Wuxi, China; 5 Department of Gynecology, Jiangnan University Medical Center, Wuxi, China; 6 Department of Clinical Research Center, Wuxi No 2 People’s Hospital, Wuxi, China; 7 Department of Clinical Research Center, Jiangnan University Medical Center, Wuxi, China; 8 Department of Urology, Wuxi No 2 People’s Hospital, Wuxi, China; 9 Department of Urology, Jiangnan University Medical Center, Wuxi, China

**Keywords:** infertility, machine learning, microbiome, multi-omics, proteomics

## Abstract

**Background:**

Infertility remains a global health challenge that often necessitates invasive and costly diagnostic procedures. While the vaginal microenvironment is known to influence reproductive health, its potential as a source of non-invasive biomarkers for infertility remains insufficiently explored. This study integrated vaginal microbiome and inflammatory proteomic profiling using machine learning to develop and validate an objective, non-invasive diagnostic model for identifying women at high risk of infertility.

**Methods:**

A total of 205 women (76 infertility patients and 129 healthy controls) were enrolled between October 2025 and February 2026. Vaginal swabs were collected for 16S rRNA sequencing and Olink Target 96 Inflammation panel analysis. LASSO, Boruta, and RFE were applied for feature selection, and eight machine learning algorithms were established to the training cohort (n = 143). Model performance was validated in the test cohort (n = 62) using AUC and DeLong tests. SHAP analysis was utilized for biological interpretability.

**Results:**

4 Olink features and 14 microbial features were retained for analysis. The microbiome-based SVM model demonstrated the highest predictive performance with an AUC of 0.815 (95% CI: 0.710–0.920), significantly outperforming the proteomics-based XGBoost model (AUC = 0.629, 95% CI: 0.489–0.768). Multi-omics based SVM model yielded an AUC of 0.799 (95% CI: 0.691–0.993). SHAP analysis identified the genera *Thomasclavelia*, unclassified *Ruminococcaceae*, and *Megamonas* as the top 3 contributing features in the predictive model. Furthermore, all Olink biomarkers retained in the prediction model, including MMP-1, MMP-10, CCL20, and CXCL5, were identified as contributing risk factors associated with infertility.

**Conclusion:**

The vaginal microbiome is a potent non-invasive biomarker for infertility, offering superior diagnostic accuracy compared to host inflammatory proteins. This machine learning-based multi-omics approach provides a promising tool for early risk stratification and personalized reproductive management.

## Introduction

Infertility, defined as failure to achieve a clinical pregnancy after ≥12 months of regular unprotected intercourse, is a common reproductive disorder affecting a substantial proportion of reproductive-age couples worldwide (Carson and Kallen, 2021; World Health Organisation Guideline Development Group for I et al., 2026a). Its causes are heterogeneous and include female, male, and unexplained factors (Carson and Kallen, 2021). Among them, female-related causes account for a large fraction of cases and involve ovulatory dysfunction, tubal or uterine pathology, endometriosis, infections, and immune dysregulation (Carson and Kallen, 2021).

Clinically, infertility often presents a more complex diagnostic landscape: even after routine evaluation of anatomy and endocrine function, a considerable subset of patients remain unexplained (World Health Organisation Guideline Development Group for I et al., 2026a). Current diagnostic pathways still rely heavily on invasive procedures (e.g., hysterosalpingography, hysteroscopy) and repeated hormonal monitoring, underscoring the need for objective, non-invasive biomarkers to enable earlier risk stratification and timely reproductive intervention (World Health Organisation Guideline Development Group for I et al., 2026a).

Emerging evidence indicates that the lower reproductive tract microenvironment, particularly the vaginal microbiome, is closely linked to fertility (Haahr et al., 2019; Serrano et al., 2019; Cheng et al., 2020; Grewal et al., 2022). In healthy reproductive-age women, the vaginal microbiota is typically dominated by *Lactobacillus* species, which help maintain a low-pH, antimicrobial milieu that limits pathogen colonization and supports sperm survival and transport (Qin et al., 2025). In contrast, dysbiosis—characterized by depletion of protective *lactobacilli* and overgrowth of anaerobic taxa including *Gardnerella*, *Prevotella*, and *Atopobium*—has been increasingly associated with adverse reproductive outcomes (Grewal et al., 2022; Lazzeri et al., 2026). While the endometrial microbiota has recently gained attention due to its proximity to the site of embryo implantation (Liu et al., 2026; Moreno and Simon, 2018), and its correlation with adverse pregnancy outcomes and impaired reproductive success has been reported (Odendaal et al., 2024; Kyono et al., 2018), the vaginal microenvironment offers distinct clinical and biological advantages. Unlike the invasive transcervical sampling required for endometrial assessment, vaginal swabs are entirely non-invasive, reducing patient discomfort and the risk of intrauterine disruption. Moreover, the vagina harbors a significantly higher microbial biomass and diversity, providing a more robust window into the host’s reproductive health (Anahtar et al., 2018). Despite these advantages, specific research investigating the direct correlation between the vaginal microbiome and infertility remains limited.

Importantly, the reproductive impact of dysbiosis is likely mediated through the host immune response (Reddy et al., 2004; Sze and Schloss, 2016). Even without overt infection, dysbiosis can induce persistent, low-grade mucosal inflammation, with altered cytokine and chemokine networks that may reduce endometrial receptivity and disrupt key reproductive processes (Reddy et al., 2004). However, traditional single-analyte assays provide an incomplete view of this complex inflammatory landscape. While 16S rRNA sequencing enables high-resolution profiling of bacterial community composition, it does not directly capture host immune status. Olink proteomics, based on Proximity Extension Assay (PEA), allows sensitive, high-throughput quantification of inflammation- and immune-related proteins from small-volume (Szeszko et al., 2026; Xue et al., 2026), minimally invasive samples such as vaginal swabs. The development of such non-invasive diagnostic tools holds profound clinical translation significance, as it enables large-scale screening and personalized management in outpatient settings.

Therefore, this study aims to characterize systematic differences in the vaginal microenvironment between women with infertility and healthy controls using non-invasive vaginal swabs. By integrating 16S rRNA sequencing with Olink targeted proteomics, and applying machine-learning methods for feature selection and model construction, we seek to develop and validate an objective, efficient, and non-invasive diagnostic model for early identification of women at high risk of infertility. Such a model may support clinical decision-making and facilitate timely, personalized fertility management.

## Methods

### Study participants

**FIGURE 1:**
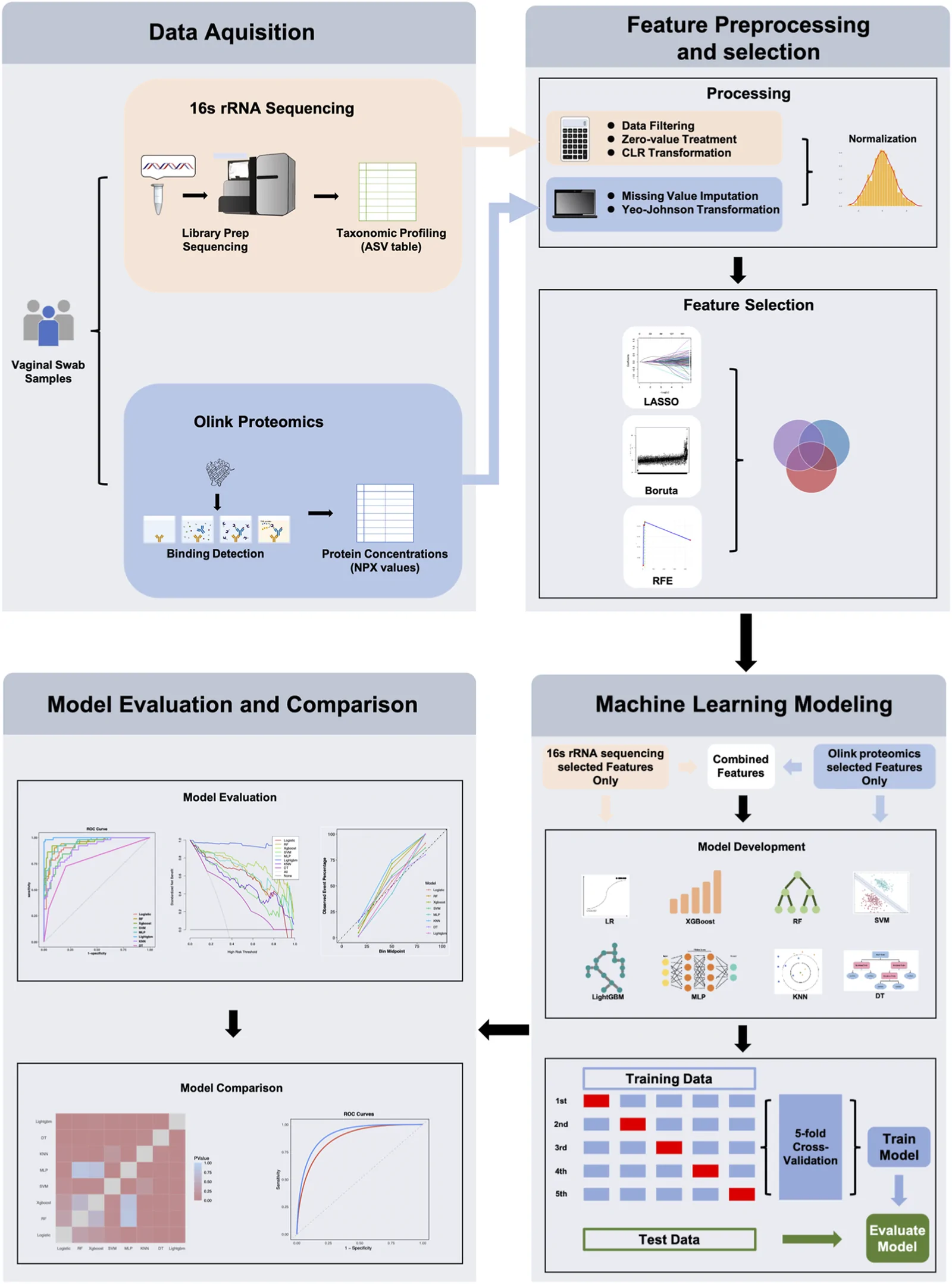
Workflow of the whole study.

A total of 205 women were enrolled in this study from the Affiliated Women’s Hospital of Jiangnan University and Jiangnan University Medical Center between October 2025 and February 2026. The study procedures were approved by the Institutional Review Board of the Affiliated Women’s Hospital of Jiangnan University (YLSL2025-095). Written informed consent was acquired from all the participants. The overall workflow is shown in Figure 1.

For the infertility group, eligible participants were required to meet all of the following inclusion criteria: age between 20 and 40 years; diagnosis of infertility, defined as failure to achieve clinical pregnancy after ≥12 months of regular unprotected intercourse with the same partner (per WHO criteria); confirmed normal semen parameters in the male partner (per WHO sixth edition reference values) to exclude a sole male-factor etiology; regular menstrual cycles (28 ± 7 days over at least three consecutive cycles); and swab sampling performed between cycle days 7 and 12 (mid-follicular phase). The control group consisted of asymptomatic healthy women with no documented history of infertility or reproductive dysfunction. While the majority of these participants had objective evidence of normal fertility (defined as at least one prior spontaneous full-term delivery with restored cycles), the group also included healthy nulligravid individuals who met all clinical criteria for normal reproductive potential. All controls were age-matched to the infertility group to minimize confounding variables.

Women with any of the following conditions were excluded from both groups: antibiotics, probiotics or hormonal medications usage within 1 month before swab sampling; vaginal douching or intravaginal medication within 1 week; the presence of irritation around the genital area or abnormal vaginal discharge within 1 week; currently menstruating or vaginal bleeding; sexual intercourse within the current menstrual cycle; any known anatomical abnormality, reproductive tract malformation, or organic cause of infertility; current use of any intrauterine device; concurrent systemic disease including diabetes mellitus or autoimmune disorders; presence of bacterial vaginosis.

Participants were randomly allocated into a training cohort and a test cohort at a 7:3 ratio (n = 143 and n = 62, respectively), with the proportion of infertility cases being 35.6% in the training cohort and 40.3% in the test cohort.

### Olink proteomics

Vaginal samples were collected during the mid-follicular phase (cycle days 7–12) using standardized sterile vaginal swabbing procedures. For each participant, six sterile vaginal swabs were collected consecutively during the same visit. Three swabs were used for microbial genomic DNA extraction and 16S rRNA sequencing, whereas the remaining three swabs were used for vaginal secretion collection and Olink Target 96 Inflammation proteomic profiling. All samples were immediately processed and stored at −80 °C until further analysis. Proteins were measured using the Olink® Target 96 Inflammation panel (Olink Proteomics AB, Uppsala, Sweden) according to the manufacturer’s instructions. The Proximity Extension Assay (PEA) technology used for the Olink protocol has been well described (Assarsson et al., 2014), and enables 92 biomarkers to be analyzed simultaneously, using 1 µL of each sample. In brief, pairs of oligonucleotide-labeled antibody probes bind to their targeted protein, and if the two probes are brought in close proximity the oligonucleotides will hybridize in a pair-wise manner. The addition of a DNA polymerase leads to a proximity-dependent DNA polymerization event, generating a unique PCR target sequence. The resulting DNA sequence is subsequently detected and quantified using a microfluidic real-time PCR instrument (Signature Q100, LC-Bio Technology CO., Ltd., Hangzhou, China). Data is then quality controlled and normalized using an internal extension control and an inter-plate control, to adjust for intra- and inter-run variation. The final assay read-out is presented in Normalized Protein Expression (NPX) values, which is an arbitrary unit on a log2-scale where a high value corresponds to a higher protein expression. Data normalization was performed using internal and external controls integrated into the Olink workflow to minimize inter-assay and intra-assay variation.

### Microbial genomic DNA extraction, 16S rRNA sequencing and data processing

Genomic DNA was extracted from vaginal swabs, and total microbial genomic DNA extraction was performed using Hexadecyl trimethyl ammonium Bromide following the manufacturer’s instructions. DNA was checked for concentration and purity at the end of the isolation protocol and stored at −80 °C until use.

The V3-V4 hypervariable region of the bacterial 16S rRNA was amplified using universal primers 341F: CCTACGGGNGGCWGCAG; 805R: GACTACHVGGGTATCTAATCC. PCR amplification was performed in a total volume of 25 μL reaction mixture containing 25 ng of template DNA, 12.5 μL PCR Premix, 2.5 μL of each primer, and PCR-grade water to adjust the volume. The PCR products were confirmed with 2% agarose gel electrophoresis. The PCR products were purifyied by AMPure XT beads (Beckman Coulter Genomics, Danvers, MA, USA) and quantified by Qubit (Invitrogen, USA). The amplicon pools were prepared for sequencing and the size and quantity of the amplicon library were assessed on Agilent 2100 Bioanalyzer (Agilent, USA) and with the Library Quantification Kit for Illumina (Kapa Biosciences, Woburn, MA, USA), respectively. The libraries were sequenced on NovaSeq PE250 platform by LC-Bio Technology Co., Ltd (Hangzhou, China).

The raw data obtained by sequencing were first quality filtered: PhiX sequences were removed, and paired-end reads with Q scores ≥20 were merged using FLASH. After dereplication using DADA2, we obtained feature table and feature sequence. Alpha diversity and beta diversity were calculated by normalized to the same sequences randomly. Then according to SILVA (release 138) classifier, feature abundance was normalized using relative abundance of each sample. Alpha diversity is applied in analyzing complexity of species diversity for a sample through five indices, including Chao1, Observed species, Goods coverage, Shannon, Simpson, and all this indices in our samples were calculated with QIIME2. Beta diversity were calculated by QIIME2, the graphs were drawn by R package.

### Feature selection and model construction

To identify the most informative and robust feature subset from the high-dimensional multiomics input space, three complementary feature selection strategies were applied independently to the training dataset, and only features consistently selected by all three methods were retained as core feature for subsequent model construction.

First, the Least Absolute Shrinkage and Selection Operator (LASSO) regression analysis method, utilizing 10-fold cross-validation, was employed to select relevant features. LASSO regularization constrained numerous uncorrelated features by setting their regression coefficients to precisely zero, determined by the regularization weight (λ). We conducted 10-fold cross-validation to identify the optimal lambda value, selecting the lambda that minimized cross-validation error. Subsequently, the features with nonzero coefficients were retained for further analysis.

Second, the Boruta algorithm was applied as a wrapper feature selection method built upon Random Forest classifiers. Boruta iteratively compares the importance of each original feature against that of randomly permuted shadow features (i.e., shuffled copies), and features that consistently demonstrate significantly higher importance than their shadow counterparts across multiple iterations are confirmed as relevant. Features classified as either “confirmed” or “tentative” by the algorithm were retained for further consideration, while those categorized as “rejected” were discarded.

Third, Recursive Feature Elimination (RFE) with a Random Forest estimator was implemented to identify the optimal feature subset through an iterative backward elimination process. At each iteration, the least important features—as ranked by classifier-derived feature importances—were pruned, and cross-validated performance was evaluated at each subset size. The feature subset yielding the highest cross-validated classification performance was selected as the RFE output.

Utilizing the selected core features, we trained eight distinct machine learning algorithms, including Support Vector Machine (SVM), Random Forest (RF), Logistical Regression (LR), Multilayer Perceptron (MLP), eXtreme Gradient Boosting (XGBoost), Light Gradient Boosting Machine (LightGBM), Decision Tree (DT), and K-Nearest Neighbors (KNN), on the training cohort and subsequently assessed their performance on the test cohorts. The classification performance was assessed by the receiver operating characteristic (ROC) curve and area under the curve (AUC). Accuracy, sensitivity, specificity, positive predictive value (PPV), negative predictive value (NPV), and F1 score were also used to assess the diagnostic performance of the models. The differences in AUCs between pairs of models were evaluated using the DeLong test.

To interpret the decision-making process of the machine learning model, we employed SHAP (SHapley Additive exPlanations) analysis based on game theory. Unlike traditional feature importance, SHAP assigns each feature an additive importance value for each specific prediction, reflecting its contribution to the deviation of the prediction from the average baseline. Global feature importance was calculated by taking the mean absolute SHAP values (mean (∣SHAP∣)) across all samples.

## Results

A total of 205 participants were enrolled in this study, comprising 129 healthy controls and 76 patients with infertility. The mean ages of the healthy and infertility groups were 32.29 ± 2.43 and 31.51 ± 3.97, respectively. Detailed data regarding pregnancy and live birth outcomes are presented in Table 1.

**TABLE 1:** Clinical characteristics of patients.

Characteristic	Healthy control (n = 129)	Infertility (n = 76)	*p* value
Age (years)			
Mean ± SD	32.29 ± 2.43	31.51 ± 3.97	0.078
Range	26–40	24–42	​
Fertility status (%)	​	​	N/A
Nulliparous	28.7	100	​
Parous	71.3	0	​
Number of pregnancies	​	​	N/A
n = 0	37	50	​
n = 1	40	14	​
n = 2	30	6	​
n = 3	15	5	​
n = 4	7	1	​
Number of live births	​	​	N/A
n = 0	56	69	​
n = 1	52	7	​
n = 2	20	0	​
n = 3	1	0	​
Etiology	​	​	N/A
Unexplained infertility	N/A	9	​
Recurrent pregnancy loss	N/A	12	​
Uterine factor	N/A	2	​
Endometriosis	N/A	4	​
Anovulation	N/A	33	​
Tubal factor	N/A	16	​

SD: standard deviation.

### Overview of vagina multi-omics profiling

#### Vaginal microbiome profiling (16S rRNA sequencing)

A total of 15,574,882 sequencing reads, ranging from 51,102 to 87,233 per sample, was generated. The sequencing reads were resolved to 7688 ASVs using DADA2, and then aggregated to two domains, 43 phyla, 93 classes, 215 orders, 369 families, 820 genera and 868 species. To ensure taxonomic reliability and focus on the primary microbial constituents, the species and domain levels were excluded from downstream analysis due to the inherent resolution limitations of the V3-V4 hypervariable region. After implementing an abundance-prevalence filter, the dataset was refined to enhance the signal-to-noise ratio. This filtration process resulted in a final set of 205 genera, 115 families, 68 orders, 35 classes, and 24 phyla.

#### Vaginal inflammatory proteomics (Olink Proteomics)

In total, all of the 92 biomarkers passed the quality control and were used in the following analysis.

#### Feature selection and core predictor identification

**FIGURE 2:**
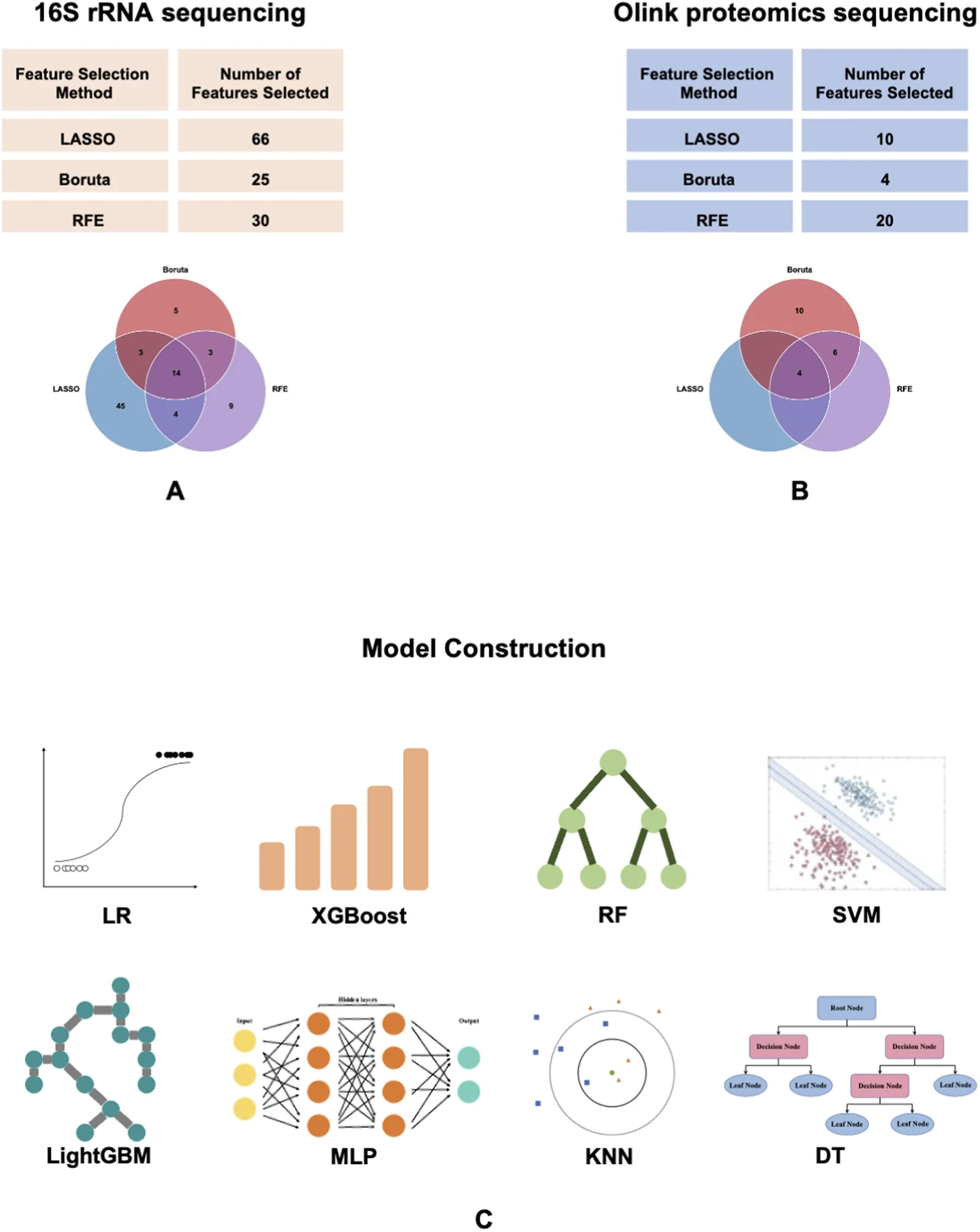
Feature selection and model construction. LASSO, Boruta and RFE were utilized to select the core feature for following analysis. **(A)** The intersection of all three methods yielded a core feature set of 14 variables in the 16S rRNA sequencing dataset. **(B)** The intersection of all three methods yielded a core feature set of four variables in the Olink proteomics dataset. **(C)** eight algorithms were utilized in model construction, including LR, XGBoost, RF, SVM, LightGBM, MLP, KNN and DT.

For 16S rRNA sequencing dataset, the LASSO identified 66 features; the Boruta algorithm retained 25 confirmed or tentative attributes; and RFE selected 30 optimal predictors. As illustrated in the Venn diagram (Figure 2A), the intersection of all three methods yielded a final core feature set of 14 variables for subsequent model construction.

For Olink proteomics dataset, the LASSO identified 10 features; the Boruta algorithm retained four confirmed or tentative attributes; and RFE selected 20 optimal predictors. As illustrated in the Venn diagram (Figure 2B), the intersection of all three methods yielded a final core feature set of four variables for subsequent model construction.

The comprehensive profiles of these final selected core features from both datasets are detailed in Supplementary Table S1.

#### Performance benchmarking of single-omics based machine learning models

**FIGURE 3:**
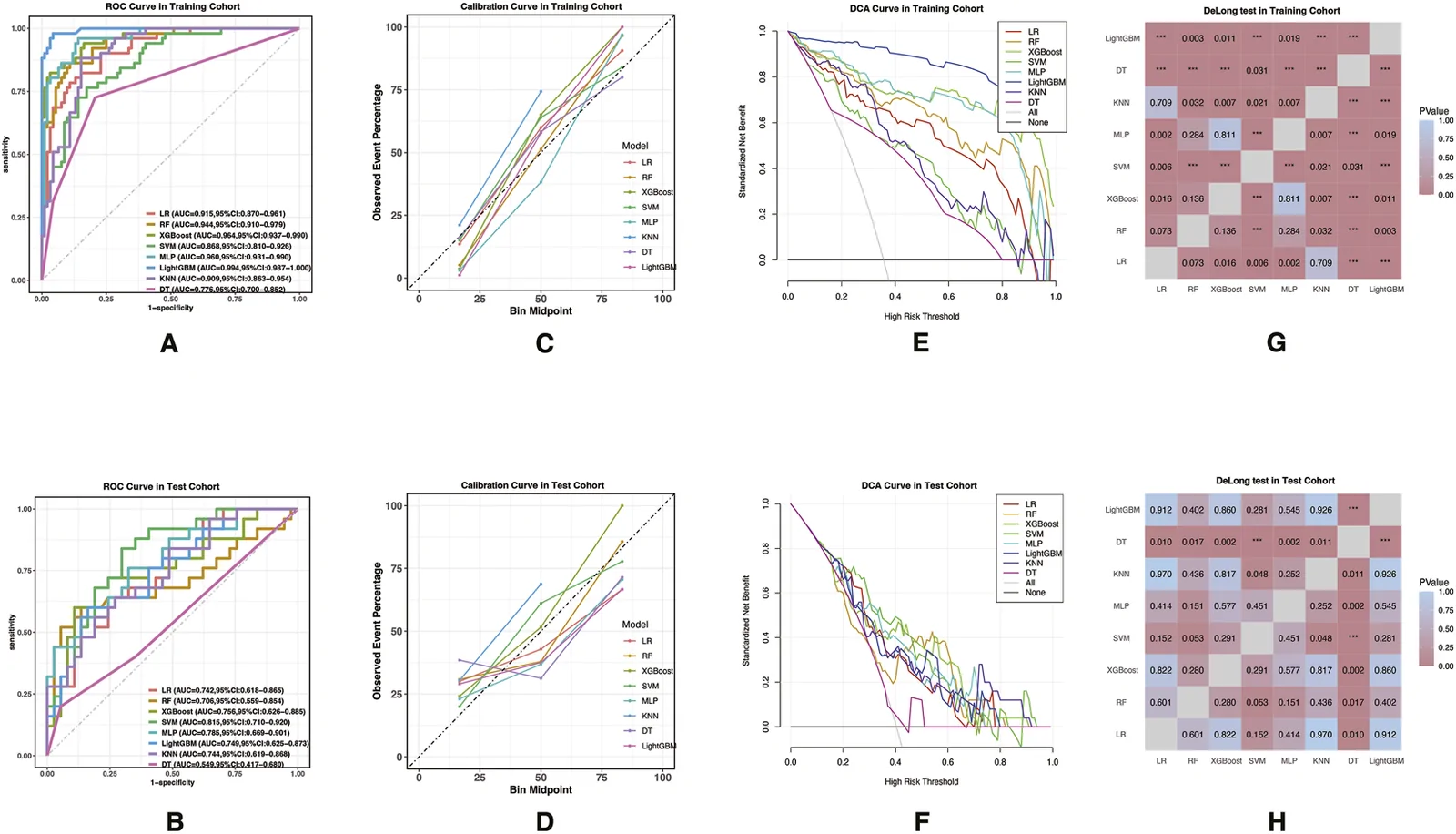
Efficacy of microboime-based models **(A,B)** The ROC curves of different machine learning models in the training and test cohort. **(C,D)** The calibration curves of different machine learning models in the training and test cohort. **(E,F)** The DCA curves of different machine learning models in the training and test cohort. **(G,H)** The heap map of AUCs comparison of different models by DeLong test in the training and test cohort.

**FIGURE 4:**
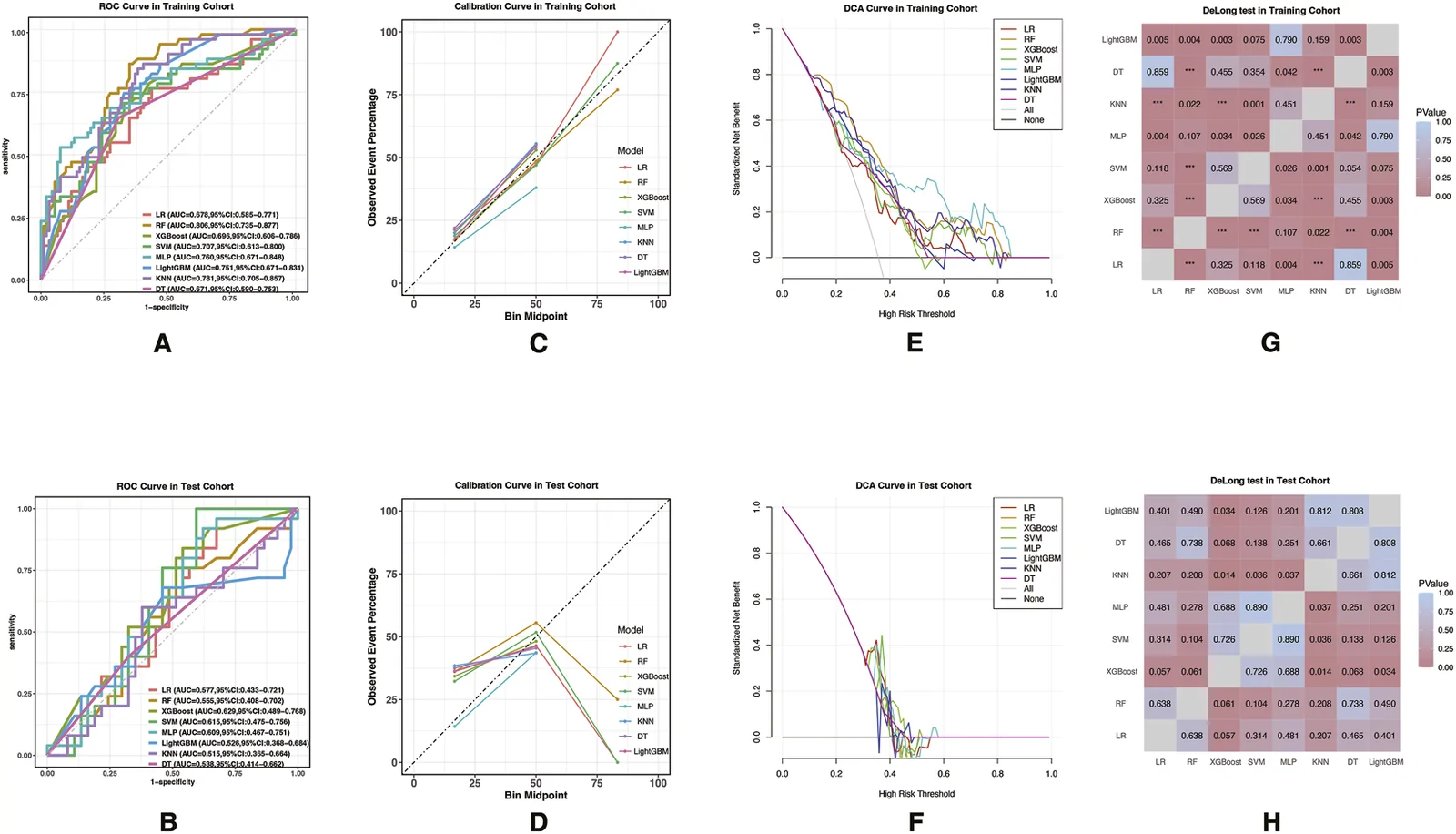
Efficacy of proteomics-based models. **(A,B)** The ROC curves of different machine learning models in the training and test cohort. **(C,D)** The calibration curves of different machine learning models in the training and test cohort. **(E,F)** The DCA curves of different machine learning models in the training and test cohort. **(G,H)** The heap map of AUCs comparison of different models by DeLong test in the training and test cohort.

To identify the most robust predictive tools, eight machine learning classifiers (LR, RF, MLP, SVM, XGBoost, LightGBM, DT, and KNN, as illustrated in Figure 2C) were independently constructed using the core features from the vaginal microbiome and proteomics features. The performances of the microbiome- and proteomics-based models are presented in Figures 3A–H and Table 2, and in Figures 4A–H and Table 3, respectively.

**TABLE 2:** AUCs for the performance of the microbiome-based predictive models.

Cohort	Classifiers	AUC	Accuracy	Sensitivity	Specificity	PPV	NPV	F1 score
Training	LR	0.915 (0.87–0.961)	0.853	0.784	0.891	0.800	0.882	0.792
	RF	0.944 (0.910–0.979)	0.881	0.863	0.891	0.815	0.921	0.838
	XGBoost	0.964 (0.937–0.990)	0.916	0.824	0.967	0.933	0.908	0.875
	SVM	0.868 (0.810–0.926)	0.804	0.765	0.826	0.709	0.864	0.736
	MLP	0.960 (0.931–0.990)	0.895	0.961	0.859	0.790	0.975	0.867
	KNN	0.909 (0.931–0.990)	0.860	0.882	0.848	0.763	0.929	0.818
	DT	0.776 (0.700–0.852)	0.769	0.725	0.793	0.661	0.839	0.692
	LightGBM	0.994 (0.987–1.000)	0.965	0.980	0.957	0.926	0.989	0.952
Test	LR	0.742 (0.618–0.865)	0.677	0.56	0.757	0.609	0.718	0.583
	RF	0.706 (0.559–0.854)	0.710	0.640	0.757	0.640	0.757	0.640
	XGBoost	0.756 (0.626–0.885)	0.742	0.600	0.838	0.714	0.756	0.652
	SVM	0.815 (0.710–0.920)	0.710	0.720	0.703	0.621	0.788	0.667
	MLP	0.785 (0.669–0.901)	0.694	0.760	0.649	0.594	0.800	0.667
	KNN	0.744 (0.619–0.868)	0.645	0.640	0.649	0.552	0.727	0.593
	DT	0.549 (0.417–0.680)	0.548	0.400	0.649	0.435	0.615	0.417
	LightGBM	0.749 (0.625–0.873)	0.645	0.640	0.649	0.552	0.727	0.593

SVM: support vector machine, LR: logistic regression, RF: random forest, KNN: k-nearest neighbor, AUC: area under the curve, NPV: negative predictive value, PPV: positive predictive value.

**TABLE 3:** AUCs for the performance of the proteomics-based predictive models.

Cohort	Classifiers	AUC	Accuracy	Sensitivity	Specificity	PPV	NPV	F1 score
Training	LR	0.678 (0.585–0.771)	0.636	0.765	0.565	0.494	0.812	0.600
	RF	0.806 (0.735–0.877)	0.727	0.863	0.652	0.579	0.896	0.693
	XGBoost	0.696 (0.606–0.786)	0.650	0.784	0.576	0.506	0.828	0.615
	SVM	0.707 (0.613–0.800)	0.678	0.745	0.641	0.535	0.819	0.623
	MLP	0.760 (0.671–0.848)	0.783	0.529	0.924	0.794	0.780	0.635
	KNN	0.781 (0.705–0.857)	0.699	0.843	0.620	0.551	0.877	0.667
	DT	0.671 (0.590–0.753)	0.678	0.647	0.696	0.541	0.780	0.589
	LightGBM	0.751 (0.671–0.831)	0.699	0.745	0.674	0.559	0.827	0.639
Test	LR	0.577 (0.433–0.721)	0.532	0.560	0.514	0.438	0.633	0.491
	RF	0.555 (0.408–0.702)	0.581	0.560	0.595	0.483	0.667	0.519
	XGBoost	0.629 (0.489–0.768)	0.565	0.640	0.514	0.471	0.679	0.542
	SVM	0.615 (0.475–0.756)	0.581	0.600	0.568	0.484	0.677	0.536
	MLP	0.609 (0.467–0.751)	0.565	0.120	0.865	0.375	0.593	0.182
	KNN	0.515 (0.365–0.664)	0.597	0.600	0.595	0.500	0.688	0.545
	DT	0.549 (0.414–0.662)	0.565	0.400	0.676	0.455	0.625	0.426
	LightGBM	0.526 (0.368–0.684)	0.565	0.480	0.622	0.462	0.639	0.471

SVM: support vector machine, LR: logistic regression, RF: random forest, KNN: k-nearest neighbor, AUC: area under the curve, NPV: negative predictive value, PPV: positive predictive value.

While the DeLong test (Figures 3G,H) indicated no statistically significant differences in AUCs among the majority of high-performing candidates in the test cohorts (with the exception of DT in the microbiome-based models, and KNN, DT, and LightGBM in the proteomics-based models), model selection was further refined by evaluating accuracy and generalization robustness.

For the microbiome-based analysis, SVM was prioritized as the optimal model due to its minimal performance gap between the training and testing sets (Supplementary Figure S1A), suggesting superior reliability, achieving an AUC of 0.815 (95% CI: 0.710–0.920), accuracy of 0.710, sensitivity of 0.720, specificity of 0.703, PPV of 0.621, NPV of 0.788, and an F1 score of 0.667.

Similarly, XGBoost was selected for the proteomics-based model for demonstrating the highest consistency between training and test accuracy (Supplementary Figure S1B). The specific performance metrics for the selected models are as follows AUC of 0.629 (95% CI: 0.489–0.768), accuracy of 0.565, sensitivity of 0.640, specificity of 0.514, PPV of 0.471, NPV of 0.679, and an F1 score of 0.542.

#### Performance of combined multi-omics diagnostic models

**FIGURE 5:**
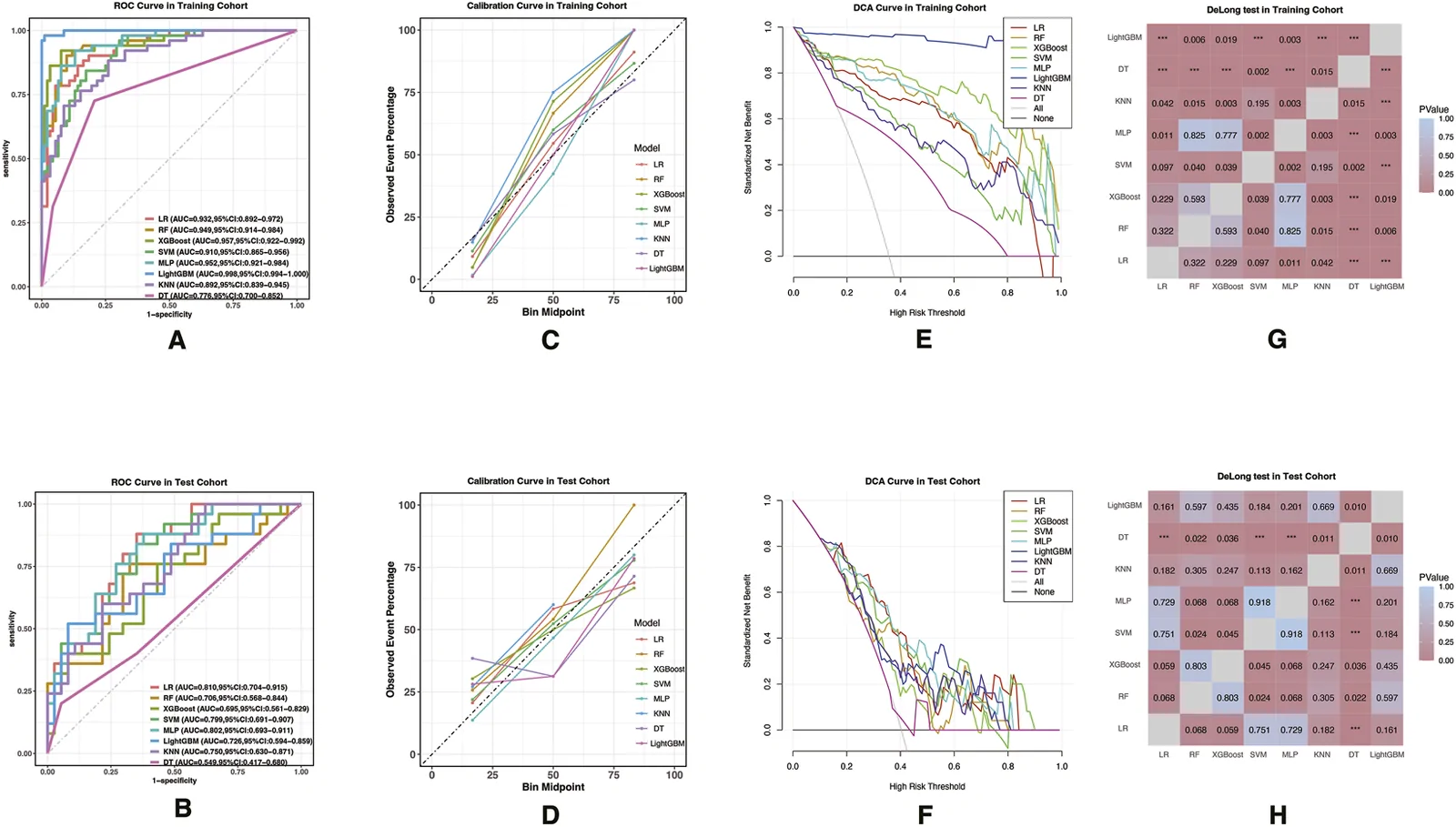
Efficacy of multiomics-based models. **(A,B)** The ROC curves of different machine learning models in the training and test cohort. **(C,D)** The calibration curves of different machine learning models in the training and test cohort. **(E,F)** The DCA curves of different machine learning models in the training and test cohort. **(G,H)** The heap map of AUCs comparison of different models by DeLong test in the training and test cohort.

To investigate whether the integration of vaginal microbiome and inflammatory proteomics profiles could synergistically enhance diagnostic performance, core features from both datasets were concatenated into a unified multi-omics panel. The same eight machine learning algorithms were applied to this integrated dataset. The performances of the Multiomics-based models are presented in Figures 5A–H and Table 4.

**TABLE 4:** AUCs for the performance of the multi-omics based predictive models.

Cohort	Classifiers	AUC	Accuracy	Sensitivity	Specificity	PPV	NPV	F1 score
Training	LR	0.932 (0.892–0.972)	0.888	0.784	0.946	0.889	0.888	0.833
	RF	0.949 (0.914–0.984)	0.902	0.902	0.902	0.836	0.943	0.868
	XGBoost	0.957 (0.922–0.992)	0.923	0.922	0.924	0.870	0.955	0.895
	SVM	0.910 (0.865–0.956)	0.832	0.843	0.826	0.729	0.905	0.782
	MLP	0.952 (0.921–0.984)	0.888	0.922	0.870	0.797	0.952	0.855
	KNN	0.892 (0.839–0.945)	0.790	0.882	0.739	0.652	0.919	0.750
	DT	0.776 (0.700–0.852)	0.769	0.725	0.793	0.661	0.839	0.692
	LightGBM	0.998 (0.994–1.000)	0.986	0.980	0.989	0.980	0.989	0.980
Test	LR	0.810 (0.704–0.915)	0.661	0.480	0.784	0.600	0.690	0.533
	RF	0.706 (0.568–0.844)	0.661	0.560	0.730	0.583	0.711	0.571
	XGBoost	0.695 (0.561–0.829)	0.613	0.480	0.703	0.522	0.667	0.500
	SVM	0.799 (0.691–0.907)	0.677	0.720	0.649	0.581	0.774	0.643
	MLP	0.802 (0.693–0.911)	0.726	0.760	0.703	0.633	0.812	0.691
	KNN	0.750 (0.630–0.871)	0.597	0.720	0.514	0.500	0.731	0.590
	DT	0.549 (0.417–0.680)	0.548	0.400	0.649	0.435	0.615	0.417
	LightGBM	0.726 (0.594–0.859)	0.694	0.560	0.784	0.636	0.725	0.596

SVM: support vector machine, LR: logistic regression, RF: random forest, KNN: k-nearest neighbor, AUC: area under the curve, NPV: negative predictive value, PPV: positive predictive value.

In the test cohort, several models, including LR, SVM, MLP, KNN, and LightGBM, demonstrated superior and comparable diagnostic efficacy. The DeLong test confirmed that there were no statistically significant differences in AUC among these top-performing classifiers (Figure 5H, *p* > 0.05). To determine the optimal model, we further evaluated their accuracy and generalization stability. While the MLP and LightGBM achieved slightly higher absolute accuracy in the test set, the SVM was ultimately selected as the final model due to its exceptional stability. The SVM exhibited the minimal discrepancy between training and test accuracy (Supplementary Figure S1C), indicating superior robustness and a lower risk of overfitting compared to other candidates. The detailed performance of the multi-omics SVM model is as follows AUC of 0.799 (95% CI: 0.691–0.993), accuracy of 0.677, sensitivity of 0.720, specificity of 0.649, PPV of 0.581, NPV of 0.774, and an F1 score of 0.643.

**FIGURE 6:**
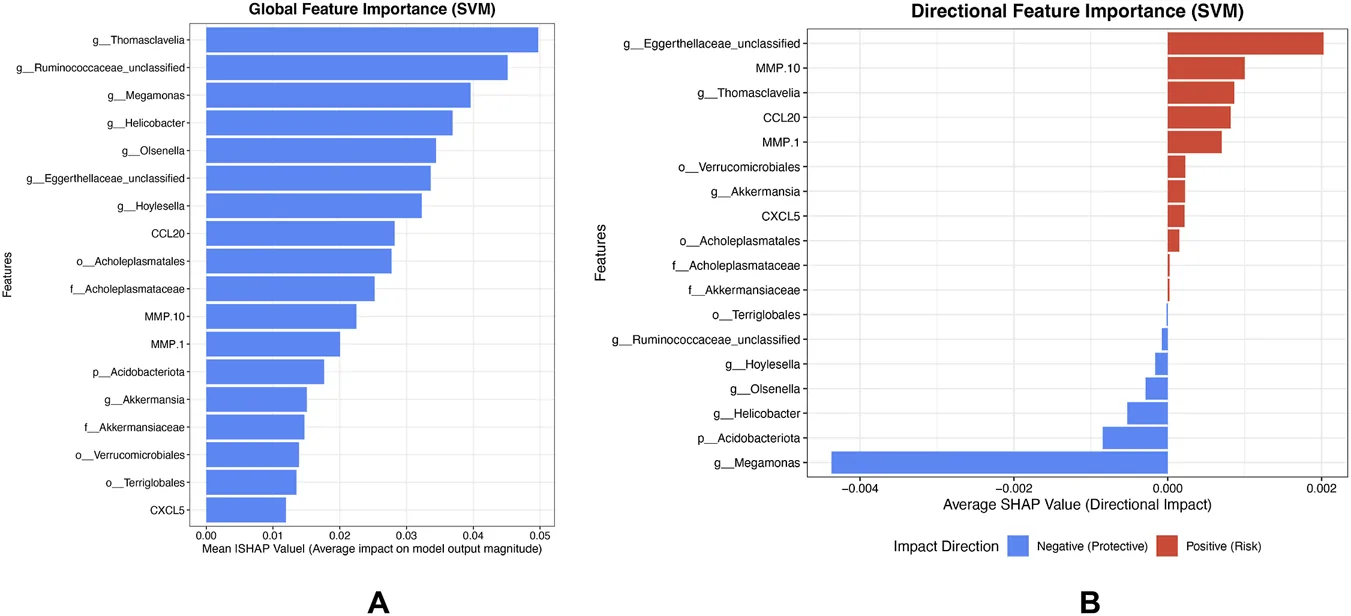
Interpretability analysis of the multi-omics diagnostic model using SHAP values **(A)** Global feature importance of the 18 core predictors, ranked by mean absolute SHAP value (mean (|SHAP|)) across all samples. **(B)** Directional SHAP summary plot showing the magnitude and direction of each feature’s contribution to model-predicted infertility risk. Red color indicates positive risk predictor and blue color indicated protective predictor.

To elucidate the contribution of individual features to the SVM model’s predictive performance, we employed SHAP analysis to calculate the global feature importance. The importance of each feature was quantified by the mean absolute SHAP value, representing its average impact on the magnitude of the model’s output. Among the 18 features, a synergistic contribution from both microbial taxa and host inflammatory proteins was observed: The genus *Thomasclavelia* emerged as the most significant predictor, exhibiting the highest mean SHAP value (Figure 6A). This was followed by the genus unclassified Ruminococcaceae and the genus *Megamonas*, which also demonstrated substantial predictive power within the model (Figure 6A). Furthermore, all Olink biomarkers retained in the prediction model, including MMP-1, MMP-10, CCL20, and CXCL5, were identified as contributing risk factors associated with infertility.

## Discussion

Whereas conventional clinical assessments for infertility emphasize anatomical patency and hormonal profiles (World Health Organisation Guideline Development Group for I et al., 2026b), the localized reproductive microenvironment frequently remains overlooked (Practice Committee of the American Society for Reproductive Medicine, 2021), this study successfully decodes the distinct signatures of the vaginal microbiota and proteome associated with infertility. By leveraging a multi-omics machine learning framework, we identified key microbial taxa and protein biomarkers that dictate the microenvironmental variations between healthy and infertile cohorts. Our predictive models demonstrate high diagnostic precision, highlighting the crucial role of the vaginal ecosystem as the first biological gate for sperm (Chen et al., 2025). These core findings present a powerful, non-invasive approach to infertility screening, shifting the diagnostic paradigm from traditional anatomical evaluations to molecular-level microenvironmental profiling.

A striking finding of our study is the distinct predictive capacities of different omics modalities and their integration. The microbiome-based SVM model (AUC = 0.815) significantly outperformed the proteomics-based XGBoost model (AUC = 0.629). Surprisingly, the integrated multiomics model (AUC = 0.799) did not yield an additive improvement over the single-omics microbiome model. From a machine learning perspective, this suggests that the host protein features provided redundant information, and their inclusion may have introduced minor noise rather than independent predictive value. Biologically, this finding underscores the paramount role of the vaginal microbiota in infertility. We hypothesize that the microbial community may function as a central ecological component that reflects or interacts with local immune responses, while inflammatory proteins could represent downstream or parallel manifestations of this microenvironmental state. From a translational perspective, this is highly encouraging: a targeted, non-invasive vaginal microbiome assay may provide sufficient diagnostic power for infertility, offering a more streamlined and cost-effective clinical tool than complex multi-omics diagnostics.

The SHAP-based interpretability framework provided a granular map of the multiomics landscape underlying infertility, identifying a robust signature of 18 key predictors across the microbiome and proteome. We observed a hierarchically consistent risk-conferring pattern, wherein both the order *Verrucomicrobiales* and its subordinate genus *Akkermansia* independently functioned as risk predictors, as did the order *Acholeplasmatales* and its affiliated family *Acholeplasmataceae*—suggesting that the risk signal within these lineages is maintained across multiple taxonomic levels rather than confined to a single rank (Figure 6B). Beyond its established role as a gut-beneficial probiotic (Ioannou et al., 2025), *Akkermansia* exhibits a striking double-edged nature (Luo et al., 2022; Grant et al., 2026), with potential disease-facilitating risks in certain contexts. Our findings underscore this specific variability, as *Akkermansia* functioned not as a protector but as a risk predictor for infertility within the reproductive tract microenvironment. Furthermore, members of the *Actinobacteriota* phylum exhibited divergent roles: the genera *Thomasclavelia* and unclassified *Eggerthellaceae* were identified as potent risk predictors. The high SHAP importance of *Thomasclavelia* is particularly noteworthy; although its specific niche in vaginal or systemic reproductive health remains to be fully defined, its strong association with infertility identifies it as a potential keystone pathogen or a highly sensitive biomarker for pathological physiological transitions.

Conversely, the model identified a distinct set of microbial features negatively associated with infertility risk. The phylum *Acidobacteriota* and its lower taxon order *Terriglobales* emerged as predictive features associated with reduced infertility risk in the machine learning model. However, given that *Acidobacteriota* is an environmentally associated bacterial lineage and is not commonly recognized as a dominant member of the vaginal microbiome, its biological interpretation should be approached cautiously and requires further validation in independent cohorts. Similarly, the genera *Olsenella* and *Hoylesella* (also within the phylum *Actinobacteriota*), along with genera *Helicobacter*, *Megamonas*, and unclassified *Ruminococcaceae*, served as protective predictors. The identification of both risk-conferring and protective genera within the same phylum (*Actinobacteriota*) underscores the necessity of genus-level analysis, as broad taxonomic classifications may mask the functional heterogeneity critical to infertility pathogenesis.

Reinforcing these microbial findings, the SHAP analysis of Olink proteomic data identified four inflammatory mediators—MMP-10, MMP-1, CCL20, and CXCL5—as consistent risk factors. The biological functions of these proteins—ranging from extracellular matrix remodeling (MMP-1 and MMP-10) (de Almeida et al., 2022) to the chemotactic recruitment of leukocytes (CCL20 and CXCL5) (Kfoury et al., 2021; Ozga et al., 2021)—align closely with the microbial risk signatures. Notably, the clinical significance of CCL20 is further underscored by studies identifying it as a potential predictive and diagnostic biomarker for preeclampsia (Wang et al., 2022), as well as its marked upregulation on the maternal aspect of the placenta in cases of spontaneous preterm birth (Akram et al., 2025). Higher expression levels of these proteins were associated with an increased model-predicted risk, providing a mechanistic link between microbial dysbiosis and a pro-inflammatory microenvironment.

### Limitations

Despite the promising performance of our models, several limitations must be acknowledged. First, the sample size of our study was relatively modest, consequently, we were unable to perform meaningful stratified analyses, limiting the model’s ability to capture rare but significant biological variations specific to distinct patient subgroups. Second, the lack of an external validation set means that the generalizability of the SVM and XGBoost models to diverse geographic or ethnic populations remains to be confirmed. Third, our multi-omics feature integration strategy relied on simple direct feature concatenation rather than more advanced multimodal fusion algorithms. Furthermore, as a cross-sectional study, we could not provide prospective validation to track the longitudinal impact of these signatures on successful conception. Future research should prioritize larger, multicenter cohorts and longitudinal designs to transition these machine learning-driven insights into robust, real-world clinical diagnostics.

## Conclusion

The vaginal microbiome is a potent non-invasive biomarker for infertility, offering superior diagnostic accuracy compared to host inflammatory proteins. This machine learning-based multi-omics approach provides a promising tool for early risk stratification and personalized reproductive management.

## Data Availability

The normalizedprotein expression units (NPX) for the Olink Target 96 Inflammation panel and taxonomy table for 16S rRNA sequencing in both infertile and healthy donors are presented in Supplementary Materials.

## Supplementary material

SUPPLEMENTARY MATERIAL FIGURE S1 Stability assessment of the machine learning models **(A)** Microbiome-based models. Comparison of stability across eight classifiers using core microbial features; SVM demonstrated the minimal accuracy gap, indicating the highest robustness. **(B)** Proteomics-based models. Stability assessment of models constructed with Olink proteomic features; XGBoost was prioritized due to its consistent performance across cohorts. **(C)** Multi-omics integration models. Evaluation of the combined diagnostic models; the SVM maintained the most stable performance, showing the least discrepancy between training and testing accuracies.

SUPPLEMENTARY TABLE S1 Selected Core features from the 16s rRNA sequencing and Olink proteomics sequencing.
