# Supplementary material for: Multi-omics integration of the vaginal microbiome and inflammatory proteome for non-invasive prediction of infertility: a machine learning approach
Source: Front Cell Dev Biol. 2026 Sep 11;14:1927235. doi: 10.3389/fcell.2026.1927235 (PMC13612421; doi:10.3389/fcell.2026.1927235)
Supplement: Supplementary file 1 [file Table1.docx]

**Table S1** selected Core features from the 16s rRNA sequencing and Olink proteomics sequencing

| Sequncing | Selected core features |
| --- | --- |
| 16S rRNA | p__Acidobacteriota |
|  | o__Acholeplasmatales |
|  | o__Terriglobales |
|  | o__Verrucomicrobiales |
|  | f__Acholeplasmataceae |
|  | f__Akkermansiaceae |
|  | g__Helicobacter |
|  | g__Olsenella |
|  | g__Megamonas |
|  | g__Thomasclavelia |
|  | g__Ruminococcaceae (unclassified) |
|  | g__Akkermansia |
|  | g__Eggerthellaceae (unclassified) |
|  | g__Hoylesella |
| Olink | CCL20 |
|  | CXCL5 |
|  | MMP-1 |
|  | MMP-10 |

CCL20: C-C motif chemokine ligand 20; CXCL5: C-X-C Motif Chemokine Ligand 5; MMP-1:Matrix Metalloproteinase 1; MMP-10: Matrix Metalloproteinase 10
